# Application of Waterborne Acrylic and Solvent-Borne Polyester Coatings on Plasma-Treated Fir (*Abies alba* M.) Wood

**DOI:** 10.3390/ma15010370

**Published:** 2022-01-04

**Authors:** Hadi Gholamiyan, Behnam Gholampoor, Reza Hosseinpourpia

**Affiliations:** 1 Department of Wood and Paper Science and Technology, Faculty of Natural Resources, University of Tehran, Karaj 77871-31587, Iran; bgholampoor@ut.ac.ir; 2 Department of Forestry and Wood Technology, Linnaeus University, Lückligs Plats 1, 35195 Vaxjo, Sweden

**Keywords:** plasma treatment, waterborne and solvent-borne coatings, adhesion strength, weathering, surface roughness

## Abstract

This research investigates the effect of plasma treatment with air, nitrogen (N_2_), and carbon dioxide (CO_2_) gases on the performance of waterborne (acrylic) and solvent-borne (polyester) coated fir (*Abies alba* M.) wood samples. The properties of the plasma-coated samples were analyzed before and after exposure to accelerated weathering and compared with those of untreated and solely treated ones. According to pull-off testing, the coating adhesion of the wood samples was considerably improved by plasma treatment, and obvious differences were observed between different plasma gases. The effect was more pronounced after the weathering test. Similar results were obtained for the abrasion resistance of the samples. The water contact angle measurement illustrated more hydrophilic character in the solely plasma-treated wood in comparison with the untreated wood. The application of coatings, however, strongly improved its hydrophobic character. The performances of waterborne and solvent-borne coatings on plasma-treated wood were comparable, although slightly better values were obtained by the waterborne system. Our results exhibit the positive effect of plasma treatment on coating performances and the increased weather resistance of the waterborne and solvent-borne coating systems on plasma-treated wood.

## 1. Introduction

Wood, as one of the oldest building materials, receives particular attention due to growing demand for sustainable buildings. Improving the performance of wood-based products by extending their service life plays a major role in the reduction of carbon footprints. Like other natural polymers, wood is susceptible to surface degradation when it is exposed to exterior conditions. The general term used to define this phenomenon is weathering, which includes photo-degradation, biodegradation, erosion by water or particles, heat and reaction to pollutants [1,2]. Photo-degradation occurs due to the extensive cleavage of wood cell components. Cleavage of the phenolic sides of lignin by absorbance of ultraviolet (UV) light is more rapid than the other wood polymers (e.g., cellulose and hemicelluloses) [3,4,5,6,7,8,9,10]. The degradation of a wood surface proceeds by removal of photo-degraded wood fragments with rain and exposure of the subjacent layers to further erosion [11,12,13].

One path to improve the surface properties of wood is through modification techniques. Among various emerging surface modification techniques, plasma treatment has been extensively used during the recent decade [4,14,15,16]. It is a dry and clean process with minor environmental concerns. The plasma state is known as the fourth state of matter [5], which can be defined as a partially ionized ambient gas consisting of ions, electrons, photons and different neutrals that may interact physically or chemically with organic matter [17,18]. Plasma treatment is a fast method and has been widely used to modify the near-surface properties of different materials, even including thermosensitive ones like wood [19,20,21,22]. Different plasma sources (e.g., radiofrequency, corona discharge and atmospheric or ionized jets), together with various inorganic gases such as nitrogen, oxygen, carbon dioxide, air and argon, have been employed previously to modify the wettability, water repellency and coating adhesion of wood and wood-based products [23,24,25]. Plasma treatment changes the near-surface chemical structure of wood by etching and introducing reactive groups [26]. The effect considerably differs in different reactor designs and plasma gases [27,28]. Klarhöfer et al. (2010) evaluated the alteration of the chemical structure of lignin and cellulose by plasma treatment in synthetic air and argon gases. The authors quoted that in an oxygen-containing atmosphere, the surface of the lignin was oxidizedl OH, CO and COOH groups were generated; and the cellulose surface was reduced by the degradation of OH groups and the formation of CO groups, while the argon atmosphere led to the degradation of polymer chains in both lignin and cellulose and the formation of CO groups [29]. Plasma pretreatment of white spruce under atmospheric pressure and oxygen gas increased the wood’s roughness and negatively affected the degree of repellency of the plasma-coated wood [23]. Riedl et al. (2014) revealed that the contact angle of water in sugar maple strongly decreased with nitrogen, argon and air-based plasma, while no significant change was observed in the wettability of maple using carbon dioxide plasma gas [27].

Several studies have evaluated the effect of different plasma treatment set-ups on the coating adhesion and weathering resistance of wood [28,29,30,31,32,33,34]. However, the majority of the results were contradictory and could not lead to a comprehensive conclusion. For instance, Riedl et al. (2014) reported that the adhesion of a waterborne, UV-curable polyurethane/polyacrylate coating on plasma-treated black spruce was significantly increased by the argon, nitrogen and air plasma ages, but no changes were detected under carbon dioxide plasma gases at different exposure times from 0.1 s to 1.5 s. Another study by Haase et al. (2019) illustrated that the glow discharge plasma treatment of black spruce did not improve its coating adhesion to water- and solvent-borne coatings, and only the resistance of the solvent-borne coatings against accelerated weathering was improved by plasma pretreatment [33]. Therefore, the present study was aimed at contributing to the understanding of wood plasma treatment at atmospheric pressure and with different plasma gases (e.g., air, nitrogen and carbon dioxide). The effect of plasma treatments on the adhesion strength of water- and solvent-borne coatings on fir wood was assessed. The coating performance and surface properties of plasma-treated and coated wood samples were also evaluated after exposure to accelerated weathering.

## 2. Materials and Methods

### 2.1. Wood Sample

Wood samples were prepared from defect- and knot-free fir sapwood (*Abies alba*) with an approximated density of 0.45 g cm^−3^. Prior to treatment, the wood samples were placed in a climate room (65 ± 5 relative humidity (RH) and 20 ± 2 °C) for 30 days. To achieve a uniform surface roughness, the surface of the wood sample was sanded with 320-grit paper before any treatment.

### 2.2. Plasma Treatment and Coatings

The plasma treatment was carried out under air, nitrogen and carbon dioxide environments using a corona treatment system (Tantec A/S, Lunderskov, Denmark). The pulse voltage was 30 kV, the pulse duration was 10–20 µs, the pulse rate frequency was 95 kHz, and the effective voltage ranged from 20 to 25 kV. Due to the high temperature gradient inside the plasma output current, the nozzle distance to the wood surface was >20 mm, and the effluent temperatures were adjusted to be below 80 °C. The plasma-treated wood samples were then coated with a 200 µm thick commercial waterborne acrylic (Akzonobel AB, Stockholm, Sweden) and solvent-borne polyester coatings (Parseshen Co., Tehran, Iran) using a film applicator (Table 1). Three replicates were used per group. The samples were then stored at 65 ± 5 RH and 20 ± 2 °C for 15 days.

### 2.3. Artificial Weathering

Artificial weathering was performed using accelerated weathering (QUV) equipment according to EN 927-6 (2018), with one cycle consisting of 150 min of UV irradiation followed by 30 min of water spraying. Over a period of 30 days, the samples were exposed to 48 cycles per week.

### 2.4. Contact Angle

The surface wettability of the treated and untreated samples before and after weathering was evaluated by a contact angle test according to the ASTM D-5946 standard (OCA 15 plus; DataPhysics Instruments GmbH Filderstadt, Germany). The probe liquid was deionized water, and the volume was 4 µm. The apparent contact angle value was recorded 5 seconds (5 s) after deposition of the water droplet. For each sample, three representative random spots were measured (*n =* 9 per group).

### 2.5. Adhesion Strength

The adhesion strengths of the coatings before and after weathering were assessed by according to ASTM D-4541 by using a tensile pull-off test (ATA Automatic—DeFelsko Corporation, Ogdensburg, NY, USA). Each sample measuring 15 × 10 × 2 (L × R × T) cm^−3^ was measured in three iterations (*n =* 9 per group) as described previously (Kielmann and Mai, 2016).

### 2.6. Surface Evaluation

The surface quality changes of the samples by means of weathering were studied by a topographic method using a confocal laser microscope (TCS SPE model- Leica Microsystems GmbH, Wetzlar, Germany). The average roughness coefficient was evaluated by LAS X control software.

### 2.7. Abrasion Resistance

The resistance against abrasion was measured using the Abraser 5135 model (Neurtek SL, Gipuzkoa, Spain) according to ASTM D4060. The sample size was 10 × 10 cm^−2^ (Tangential direction). Three replicates were prepared from each sample type (*n =* 9 per group). The samples were abraded with S-42 sandpaper with approximately 3 min^−1^ for 250 revolutions. The wear index (I) of each sample was calculated according to Equation (1). The lower the wear index, the better the abrasion resistance [35]:

(1) $$I=\frac{\left(a-b\right)1000}{c}\times 100$$

where *a* and *b* are the mass of specimens before and after abrasion (g), respectively, and *c* is the number of test cycles.

### 2.8. Statistical Test

The results were analyzed by one-way analysis of variance (ANOVA) at a 95% confidence interval (*p* < 0.05) using the SPSS version 16 statistical software package (IBM Corp., Armonk, NY, USA), as described previously [36]. The statistical differences between the values were assessed by the Duncan test.

## 3. Results and Discussion

### 3.1. Surface Hydrophobization

**Figure 1:**
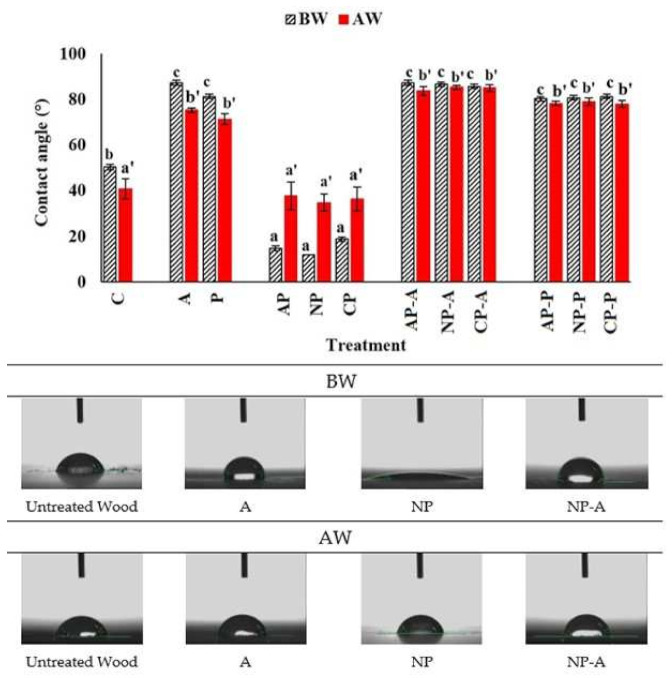
Contact angle of water on untreated, plasma-treated and coated samples before and after weathering. The statistically differences were tested with ANOVA and Duncan test. Values labeled with the same letter were statistically equal at an error probability of α = 0.05. Error bars represent standard deviations.

The contact angle measurement was performed to analyze the hydrophobic character of the untreated and treated wood surfaces before (BW) and after (AW) weathering (Figure 1). As was expected, the contact angle values of the samples slightly decreased after weathering. The untreated wood displayed a hydrophilic character by having a contact angle value of 49°. This was in agreement with the previous findings [37,38]. The samples coated with the waterborne acrylic showed a higher mean contact angle value than the solvent-borne polyester coating. Plasma treatment, however, considerably decreased the contact angle value, which indicated the more hydrophilic surface. The lowest contact angle value of 12° was recorded in plasma-treated wood with nitrogen gas, while the differences between various plasma gases were statistically insignificant. This could be related to the alteration of the surface energy of the wood due to oxidation of the wood substances [39,40]. Although the plasma-treated and subsequently waterborne coated samples showed higher contact angle values in comparison with the other treatments, the values were statistically not significant. The etched wood surface under plasma treatment was described previously as a desired phenomenon to increase the surface wettability for better coating penetration [33].

### 3.2. Adhesion Quality

**Figure 2:**
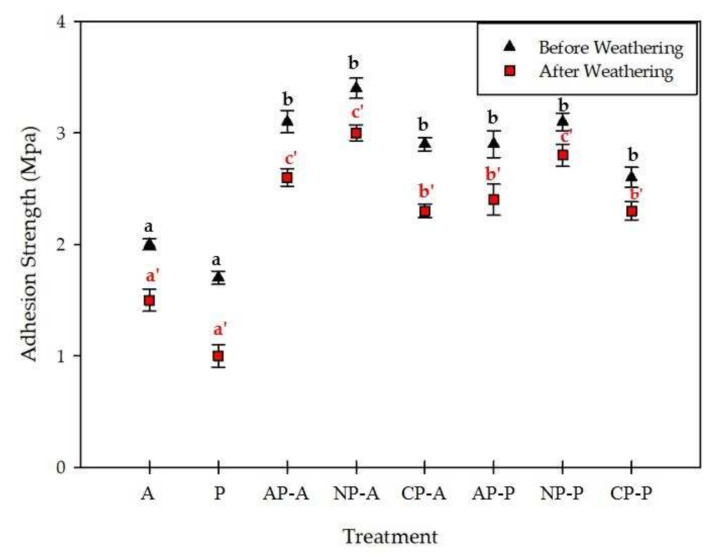
Adhesion strengths of untreated, plasma-treated and coated samples before and after weathering. The indicators were statistically tested with ANOVA and Duncan test at a 95% confidence interval (*p* < 0.05). Error bars represent standard deviations.

The adhesion strengths of the coatings on the fir wood samples as a function of the plasma treatment were evaluated before and after weathering (Figure 2). The adhesion of the waterborne and solvent-borne coatings on the untreated and plasma-treated wood samples decreased considerably after weathering. Plasma treatment, however, significantly improved the adhesion of the coating films to the wood samples, and the effect of various plasma gases was also pronounced. Plasma treatment with nitrogen gas exhibited the highest adhesion of the coating films to the wood samples before and after weathering (NP-A and NP-P). This was followed by plasma treatment with air (oxygen). The positive effect of plasma treatment on increasing coating adhesion to the wood surface was repeatedly reported previously, and it seemed to be related to the increasing interaction between the wood and coating films either through mechanical locking (i.e., via physical etching of the wood surface) or chemical bonding [41,42,43,44,45,46]. The compatibility of the waterborne acrylic coating with the plasma-treated samples was higher than that of the solvent-borne ones, although the differences were statistically insignificant. This could be attributed to greater penetration of the polar solvent-based coating (e.g., water) into the wood structure. The lowest adhesion was observed in the plasma-treated samples with CO_2_ gas and coated with a solvent-borne coating (CP-P). Riedl et al. (2014) also quoted a similar effect on the coating adhesion of black spruce after plasma treatment with CO_2_ [27].

### 3.3. Surface Roughness

**Figure 3:**
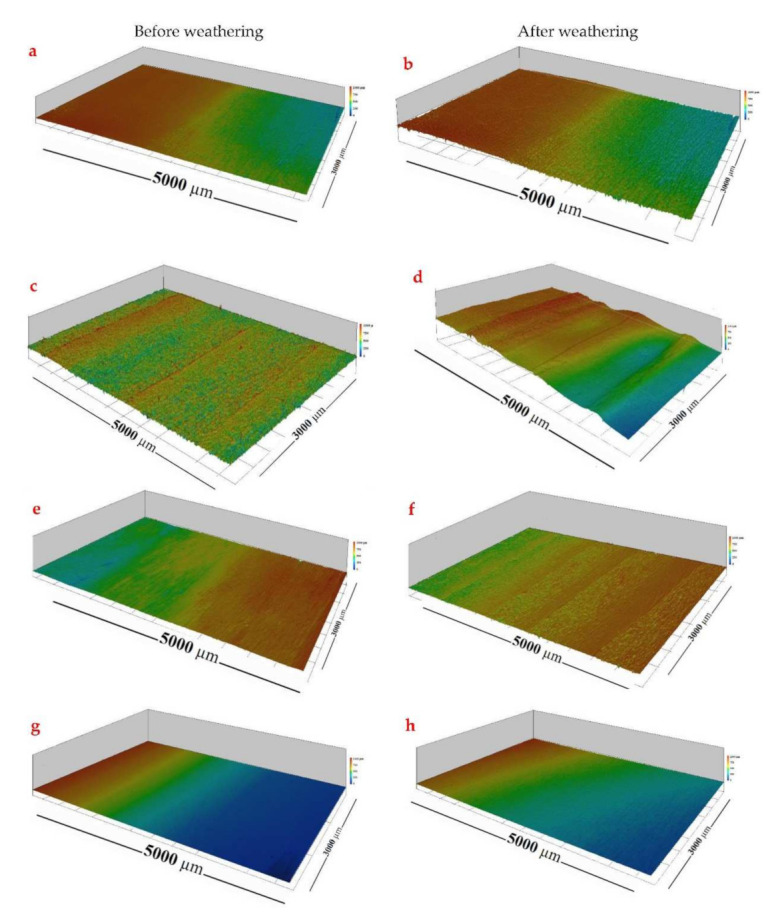
Laser scanning confocal microscope images of untreated wood (**a**,**b**), NP (**c**,**d**), A (**e**,**f**) and NP-A (**g**,**h**) before and after weathering.

**Figure 4:**
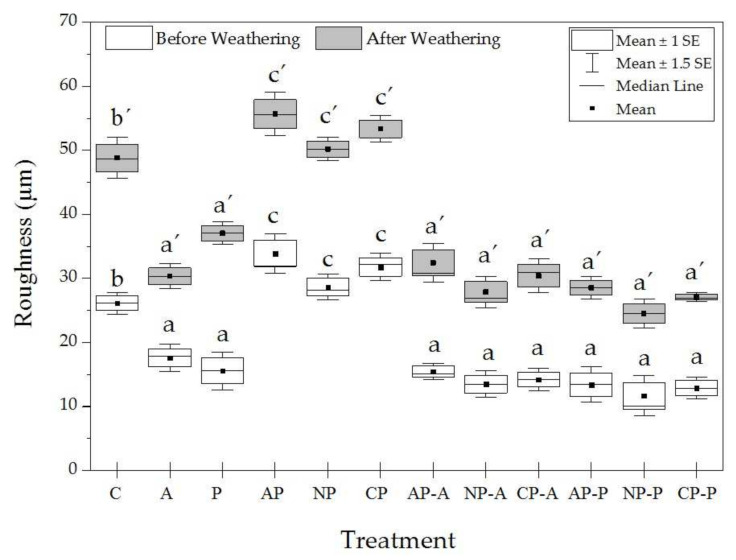
Surface roughness of untreated and treated wood samples. The indicators were statistically tested with ANOVA and Duncan test. The indicators were statistically differences were tested with ANOVA and Duncan test. Values labeled with the same letter were statistically equal at an error probability of α = 0.05. Error bars represent standard deviations.

The laser scanning confocal microscope (LSCM) is considered a flexible and versatile optical technique for evaluating the surface topography [42]. The surface topographies of the untreated, plasma-treated and plasma-treated coated samples before and after weathering are illustrated in Figure 3. The roughness values obtained from LSCM analysis are also shown in Figure 3. Plasma treatment of wood with air, nitrogen and CO_2_ gases significantly increased the surface roughness by 29.9%, 9.6% and 21.4%, respectively (Figure 4). This might be due to the etching of the wood surfaces by the plasma treatment. The wood coatings provided smoother surfaces, while as can be seen in Figure 4, the roughness values were slightly lower in the samples with plasma treatment and solvent-borne coatings (i.e., the lowest roughness value of 11.6 µm was obtained in wood plasma-treated with nitrogen and coated with a solvent-borne polyester (NP-P) sample). The weathering strongly increased the surface roughness of the wood samples, and the highest increment in the surface roughness was observed in the untreated and uncoated plasma-treated samples (Figure 3d). The effect of different plasma gases on the surface roughness was statistically insignificant (α = 0.05). The plasma-treated and coated wood samples were more stable after weathering (Figure 3h). As reported by Haase et al. (2019), the plasma treatment opened the pits that connect the wood fibers, and this effect may explain the higher adhesion strength of the coatings to the surface of the plasma-treated wood [33]. Our results also show that the surface roughness increased considerably under plasma treatment. This suggests that aside from the possible chemical interaction of coatings with plasma-treated wood, there was mechanical interlocking of the coatings in the structure of treated wood due to the increased wood surface.

### 3.4. Abrasion Resistance

**Figure 5:**
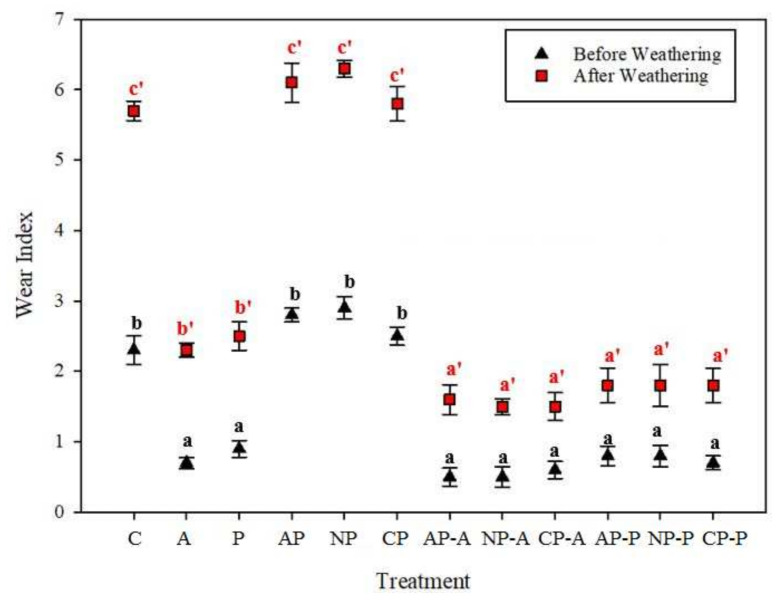
Wear index of untreated and treated samples before and after weathering. The indicators were statistically tested with ANOVA and Duncan test at at a 95% confidence interval (*p* < 0.05). Error bars represent standard deviations.

**Figure 6:**
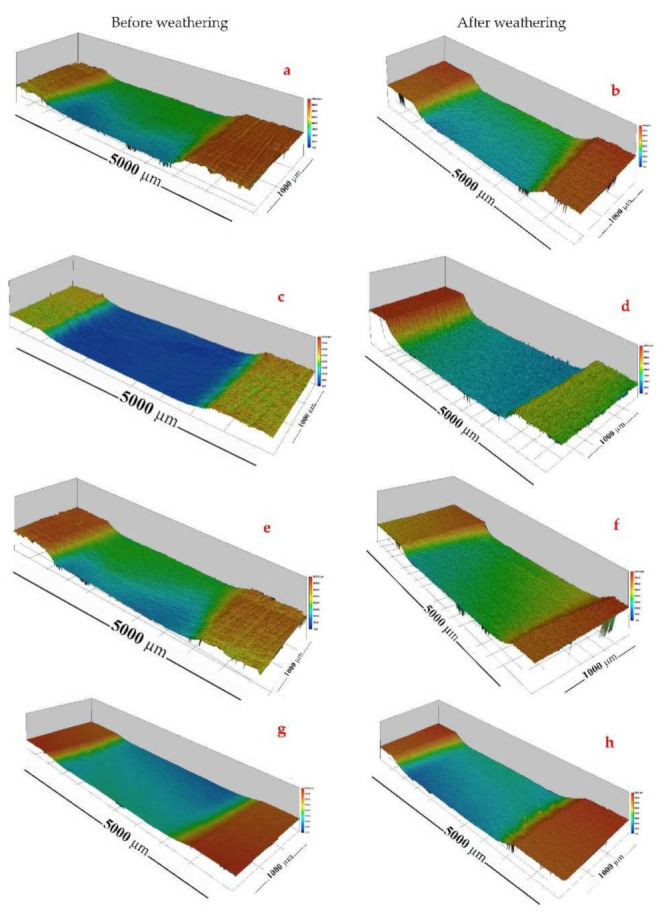
Laser scanning confocal microscope images of untreated wood (**a,b**), NP (**c,d**), A (**e,f**) and NP-A (**g,h**) before and after artificial weathering.

The wear index (WI) of the wood samples as a function of the plasma treatment and coatings was evaluated by the mass changes during the abrasion test (Figure 5). Laser scanning confocal microscopy was also employed to visualize the wood surface topography before and after the artificial weathering (Figure 6). Significant changes were observed in the different treated samples. The highest mass loss was observed in the plasma-treated samples (Figure 5) followed by the untreated wood (Figure 5). The changes were obvious by LSCM images (Figure 6a–h). The abrasion resistance of the samples was enhanced significantly by the coatings, as indicated by the lower wear index (WI) value. The waterborne coating showed a slightly higher resistance to abrasion than the solvent-borne one. These results are consistent with those of Pavlic et al. (2021), who reported that the abrasion resistance of wood was enhanced significantly by waterborne coatings [44]. The lowest WI values were obtained in the plasma-coated wood samples, which illustrated a better abrasion resistance. This is apparent in Figure 6g with a decreasing abrasion depth. The differences in the WI of different plasma-coated samples were not statistically significant. A similar trend was also observed after artificially weathering the samples, although the WI values were significantly higher compared with the unweathered ones. More details on the WI change of the samples due to artificial weathering can be found in the Supplementary Materials. It is evident by the results that less material was removed from the plasma-treated and subsequently coated samples. This could be attributed to the deeper penetration of the coating film applied on a wood structure [44]. A greater adhesion of the coating films to the wood substrates, as indicated in Figure 2, could be an additional reason [45,46].

## 4. Conclusions

Waterborne and solvent-borne coatings were successfully applied on plasma-treated fir wood samples. The surface roughness of the wood samples was increased by plasma treatment, although no meaningful differences were observed under different plasma gases. The plasma-coated wood showed a higher adhesion strength and abrasion resistance and a more hydrophobic surface than the untreated and solely treated ones before and after weathering. In conclusion, the ability of plasma treatment to improve the coating performance on fir wood seems to depend on its effect on the surface roughness of the wood. Future research should focus on the outdoor weathering resistance of plasma-coated wood. Evaluating the possible chemical reaction between plasma-treated wood and coating films would be also useful.

## Figures and Tables

**Table 1:** Plasma treatment and coating formulations.

Coating Formulation	Plasma Environment	Sample Code
-	-	C
Acrylic	-	A
Polyester	-	P
-	Air	AP
-	Nitrogen	NP
-	CO_2_	CP
Acrylic	Air	AP-A
Acrylic	Nitrogen	NP-A
Acrylic	CO_2_	CP-A
Polyester	Air	AP-P
Polyester	Nitrogen	NP-P
Polyester	CO_2_	CP-P

## Data Availability

The data presented in this study are available on request from the corresponding author.

## Supplementary Materials

The following supporting information can be downloaded at: https://www.mdpi.com/article/10.3390/ma15010370/s1, Table S1: WI changes of the untreated and treated samples by artificial weathering.

## References

[1] Žigon J., Kovač J., Saražin J., Šernek M., Petrič M., Dahle S. (2020). Enhancement of strength of adhesive bond between wood and metal using atmospheric plasma treatment. Cellulose.

[2] Cogulet P., Blanchet V., Landry and P. (2018). Morris, Weathering of wood coated with semi-clear coating: Study of interactions between photo and biodegradation. Int. Biodeterior. Biodegrad..

[3] Xie L., Tang Z., Jiang L., Breedveld V., Hess D.W. (2015). Creation of superhydrophobic wood surfaces by plasma etching and thin-film deposition. Surf. Coat. Technol..

[4] De Cademartori P.H.G., Stafford L., Blanchet P., Magalhães W.L.E., de Muniz G.I.B. (2017). Enhancing the water repellency of wood surfaces by atmospheric pressure cold plasma deposition of fluorocarbon film. RSC Adv..

[5] Haase J.G., Leung L.H., Evans P.D. (2019). Plasma pre-treatments to improve the weather resistance of polyurethane coatings on black spruce wood. Coatings.

[6] Sauerbier P., Anderson J., Gardner D.J. (2018). Surface Preparation and Treatment for Large-Scale 3D-Printed Composite Tooling Coating Adhesion. Coatings.

[7] Evans P., Thay P.D., Schmalzl K. (1996). Degradation of wood surfaces during natural weathering. Effects on lignin and cellulose and on the adhesion of acrylic latex primers. Wood Sci. Technol..

[8] McNally A., Moody E., McNeill K. (2005). Kinetics and mechanism of the sensitized photodegradation of lignin model compounds. Photochem. Photobiol. Sci..

[9] Cogulet A., Blanchet P., Landry V. (2016). Wood degradation under UV irradiation: A lignin characterization. J. Photochem. Photobiol..

[10] Kielmann B., Butter K., Mai C. (2017). Modification of wood with formulations of phenolic resin and iron-tannin-complexes to improve material properties and expand colour variety. Eur. J. Wood Wood Prod..

[11] Yamamoto A., Kymäläinen M., Lindroos T., Rohumaa A., Sokka K., Rautkari L. (2017). Surface activation of wood by corona treatment and NaOH soaking for improved bond performance in plywood. BioResources.

[12] Chen W., Zhou X., Zhang X., Bian J., Shi S., Nguyen T., Chen M., Wan J. (2017). Fast enhancement on hydrophobicity of poplar wood surface using low-pressure dielectric barrier discharges (DBD) plasma. Appl. Surf. Sci..

[13] Hon D.N.S., Hon D.N.S., Shiraishi N. (2001). Weathering and Photochemistry of Wood. Wood and Cellulose Chemistry.

[14] Youssefi R., Maier J., Scheffknecht G. (2020). Experimental investigations on plasma-assisted wood pellet ignition for the start-up of biomass-fired power stations. Renew. Sust. Energy Rev..

[15] Gascón-Garrido P., Thévenon M.F., Mainusch N., Militz H., Viöl W., Mai C. (2017). Siloxane-treated and copper-plasma-coated wood: Resistance to the blue stain fungus *Aureobasidium pullulans* and the termite *Reticulitermes flavipes*. Int. Biodeterior..

[16] Yasuda H. (1985). Plasma Polymerization.

[17] Becker K.H., Belkind A. (2003). Introduction to Plasmas. Vac. Technol. Coat..

[18] Ren C.S., Wang K., Nie Q.Y., Wang D.-Z., Guo S.-H. (2008). Surface modification of PE film by DBD plasma in air. Appl. Surf. Sci..

[19] Feddes B., Wolke J.G.C., Vredenberg A.M., Jansen J.A. (2004). Adhesion of calcium phosphate ceramic on polyethylene (PE) and polytetrafluoroethylene (PTFE). Surf. Coat. Technol..

[20] Noeske M., Degenhardt J., Strudthoff S., Lommatzsch U. (2004). Plasma jet treatment of five polymers at atmospheric pressure: Surface modifications and the relevance for adhesion. Int. J. Adhes..

[21] Lehocký M., Drnovská H., Lapčíková B., Barros-Timmons A.M., Trindade T., Zembala M., Lapčík L. (2003). Plasma surface modification of polyethylene. Colloids Surf. A Physicochem. Eng. Asp..

[22] Hazir E., Seker S., Koc K.H., Dilik T., Erdinler E.S., Ozturk E. (2021). Optimization of plasma treatment parameters to improve the wood-coating adhesion strength using Taguchi integrated desirability function approach. J. Adhes. Sci. Technol..

[23] Yáñez-Pacios A.J., Martín-Martínez J.M. (2017). Surface modification and improved adhesion of wood-plastic composites (WPCs) made with different polymers by treatment with atmospheric pressure rotating plasma jet. Int. J. Adhes. Adhes..

[24] Busnel F., Blanchard B., Prégent J., Stafford L., Riedl B., Blanchet P., Sarkissian A. (2010). Modification of sugar maple (*Acer saccharum*) and black spruce (*Picea mariana*) wood surfaces in a dielectric barrier discharge (DBD) at atmospheric pressure. J. Adhes. Sci. Technol..

[25] Riedl B., Angel C., Prégent J., Blanchet P., Stafford L. (2014). Effect of wood surface modification by atmospheric-pressure plasma on waterborne coating adhesion. BioResources.

[26] Klarhöfer L., Viöl W., Maus-Friedrichs W. (2010). Electron spectroscopy on plasma treated lignin and cellulose. Holzforschung.

[27] Blanchard V., Blanchet P., Riedl B. (2009). Surface energy modification by radiofrequency inductive and capacitive plasma at low pressures on sugar maple: An exploratory study. Wood Fiber Sci..

[28] Blanchard V., Stirling R. (2013). Plasma pre-treatment enhances field performance of exterior wood coatings. Wood Fiber Sci..

[29] Van den Bulcke J., Van Acker J., Stevens M. (2008). Experimental and theoretical behavior of exterior wood coatings subjected to artificial weathering. J. Coat. Technol. Res..

[30] Rijckaert V., Stevens M., Van Acker J. (2001). Effect of some formulation parameters on the penetration and adhesion of water-borne primers into wood. Eur. J. Wood Wood Prod..

[31] Kielmann B.C., Mai C. (2016). Application and artificial weathering performance of translucent coatings on resin-treated and dye-stained beech-wood. Prog. Org. Coat..

[32] Nejad M., Shafaghi R., Ali H., Cooper P. (2013). Coating performance on oil-heat treated wood for flooring. BioResources.

[33] Hosseinpourpia R., Adamopoulos S., Walther T., Naydenov V. (2020). Hydrophobic formulations based on tall oil distillation products for high-density fiberboards. Materials.

[34] Molnár Z., Magoss E., Fuchs I., Csiha C. (2018). Stability of thermosmoothed and precision planed solid wood surfaces. Eur. J. Wood Prod..

[35] Jankowska A., Zbieć M., Kozakiewicz P., Koczan G., Oleńska S., Beer P. (2018). The wettability and surface free energy of sawn, sliced and sanded European oak wood. Maderas Cienc. Tecnol..

[36] Sakata I., Morita M., Tsuruta N., Morita K. (1993). Activation of wood surface by corona treatment to improve adhesive bonding. J. Appl. Polym. Sci..

[37] Avramidis G., Klarhöfer L., Maus-Friedrichs W., Militz H., Viöl W. (2012). Influence of air plasma treatment at atmospheric pressure on wood extractives. Polym. Degrad. Stab..

[38] Gholamiyan H. (2020). Plasma Modification to Improve the Adhesion Resistance of the Wood Coating. JSCT.

[39] Peng X.R., Zhang Z.K. (2019). Improvement of paint adhesion of environmentally friendly paint film on wood surface by plasma treatment. Prog. Org. Coat..

[40] Sauerbier P., Köhler R., Renner G., Militz H. (2020). Plasma Treatment of Polypropylene-Based Wood–Plastic Composites (WPC): Influences of Working Gas. Polymers.

[41] Bahners T., Gutmann J.S. (2012). Photo-initiated lamination of polyethylene (PE) and poly (ethylene terephthalate) (PET). J. Adhes. Sci. Technol..

[42] Švorčík V., Kolářová K., Slepička P., Macková A., Novotná M., Hnatowicz V. (2006). Modification of surface properties of high and low density polyethylene by Ar plasma discharge. Polym. Degrad. Stab..

[43] Bezák T., Kusý M., Eliáš M., Kopček M. Surface roughness determination using laser scanning confocal microscope Zeiss LSM 700, METAL 2013. Proceedings of the 22nd International Conference on Metallurgy and Materials.

[44] Pavlic M., Petric M., Žigon J. (2021). Interactions of Coating and Wood Flooring Surface System Properties. Coatings.

[45] Žigon J., Dahle S., Petrič M., Pavlič M. (2020). Enhanced Abrasion Resistance of Coated Particleboard Pre-Treated with Atmospheric Plasma. Drv. Ind. Znan. Čas. Pitanja Drvne Tehnol..

[46] Jnido G., Ohms G., Viöl W. (2020). One-Step Deposition of Polyester/TiO2 Coatings by Atmospheric Pressure Plasma Jet on Wood Surfaces for UV and Moisture Protection. Coatings.

