# Supplementary material for: Application of Waterborne Acrylic and Solvent-Borne Polyester Coatings on Plasma-Treated Fir (Abies alba M.) Wood
Source: Materials (Basel). 2022 Jan 4;15(1):370. doi: 10.3390/ma15010370 (PMC8745835; doi:10.3390/ma15010370)
Supplement: Supplementary file 1 [file materials-15-00370-s001.zip › materials-1530563-supplementary.pdf]

materials

MDPI

Supplementary Materials

Application of Waterborne Acrylic and Solvent-Borne Polyester Coatings on Plasma-Treated Fir (*Abies alba* M.) Wood

Hadi Gholamiyan [1,*], Behnam Gholampoor [1] and Reza Hosseinpourpia [2,*]

[1] Department of Wood and Paper Science and Technology, Faculty of Natural Resources, University of Tehran, 77871-31587 Karaj, Iran; hadi_gholamiyan@ut.ac.ir (H.G.); bgholampoor@ut.ac.ir (B.G.)

[2] Department of Forestry and Wood Technology, Linnaeus University, Lückligs Plats 1, 35195 Vaxjo, Sweden; reza.hosseinpourpia@lnu.se (R.H.)

* Correspondence: hadi_gholamiyan@ut.ac.ir (H.G.); reza.hosseinpourpia@lnu.se (R.H.)

Citation: Gholamiyan, H.; Gholampoor, B.; Hosseinpourpia, R. Application of Waterborne Acrylic and Solvent-Borne Polyester Coatings on Plasma-Treated Fir (*Abies alba* M.) Wood. *Materials* **2022**, *15*, 370. https://doi.org/10.3390/ma15010370

Academic Editor: Jānis Andersons

Received: 13 December 2021
Accepted: 27 December 2021
Published: 4 January 2022

Publisher's Note: MDPI stays neutral with regard to jurisdictional claims in published maps and institutional affiliations.

Copyright: © 2022 by the authors. Licensee MDPI, Basel, Switzerland. This article is an open access article distributed under the terms and conditions of the Creative Commons Attribution (CC BY) license (https://creativecommons.org/licenses/by/4.0/).

Wear Index changes

Wear index (WI) changes of untreated and treated wood samples as a function ofartificial weathering was calculated as follow:

$$\alpha = \frac{WI_i - WI_j}{WI_j} \times 100$$

where α is a wear index changes, WI_i is a wear index after weathering and WI_j is the wear index before weathering.

Table S1. WI changes of the untreated and treated samples by artificial weathering.

Treatment	α (%)
C	152,2
A	214,3
P	166,7
AP	130,8
NP	121,4
CP	141,7
AP-A	200,0
NP-A	160,0
CP-A	133,3
AP-P	112,5
NP-P	112,5
CP-P	142,9
